# How Thoughts Give Rise to Action - Conscious Motor Intention Increases the Excitability of Target-Specific Motor Circuits

**DOI:** 10.1371/journal.pone.0083845

**Published:** 2013-12-26

**Authors:** Volker R. Zschorlich, Rüdiger Köhling

**Affiliations:** 1 Department of Movement Science, University of Rostock, Rostock, Germany; 2 Interdisciplinary Faculty - Department of Aging Science, University of Rostock, Rostock, Germany; 3 Faculty of Medicine, University of Rostock, Rostock, Germany; 4 Faculty of Philosophy, University of Rostock, Rostock, Germany; 5 Oscar-Langendorff-Institute of Physiology, University of Rostock, Rostock, Germany; IIT - Italian Institute of Technology, Italy

## Abstract

The present study shows evidence for conscious motor intention in motor preparation prior to movement execution. We demonstrate that conscious motor intention of directed movement, combined with minimally supra-threshold transcranial magnetic stimulation (TMS) of the motor cortex, determines the direction and the force of resulting movements, whilst a lack of intention results in weak and omni-directed muscle activation.

We investigated changes of consciously intended goal directed movements by analyzing amplitudes of motor-evoked potentials of the forearm muscle, flexor carpi radialis (FCR), and extensor carpi radialis (ECR), induced by transcranial magnetic stimulation over the right motor cortex and their motor outcome. Right-handed subjects were asked to develop a strong intention to move their left wrist (flexion or extension), without any overt motor output at the wrist, prior to brain stimulation.

Our analyses of hand acceleration and electromyography showed that during the strong motor intention of wrist flexion movement, it evoked motor potential responses that were significantly larger in the FCR muscle than in the ECR, whilst the opposite was true for an extension movement. The acceleration data on flexion/extension corresponded to this finding. Under no-intention conditions again, which served as a reference for motor evoked potentials, brain stimulation resulted in undirected and minimally simultaneous extension/flexion innervation and virtually no movement.

These results indicate that conscious intentions govern motor function, which in turn shows that a neuronal activation representing an “intention network” in the human brain pre-exists, and that it functionally represents target specific motor circuits. Until today, it was unclear whether conscious motor intention exists prior to movement, or whether the brain constructs such an intention after movement initiation. Our study gives evidence that motor intentions become aware before any motor execution.

## Introduction

Movement preparation in voluntary movement is a complex process of different cooperating brain areas. Motor preparation can be more or less automatic or under voluntary control. In the last case, one part of this process is a goal-directed movement intention. Both in the fields of psychology [1]–[3], and basic neurosciences [4]–[7], the processes of goal-directed voluntary movement were studied intensely. In this context, closely related brain functions, such as attention and intention, were locally distinguishable from each other by functional brain imaging [4], [8]. Likewise, Gallivan et al. [9] were able to decode movement intentions from brain signals by functional magnetic resonance imaging (fMRI) pattern recognition techniques. Moreover, it is known that cognition exerts a modulating influence on motor-evoked potentials (MEP). This modulation relates, i.e. to mental imagery [10], [11], to external stimuli [11], [12], and even to crossmodal phenomena [14], [15].

In the rapidly developing field of brain-computer-interfaces, the knowledge of functional mechanisms, and the properties of coupling between motor intention [16], [17] and motor act, is of particular importance. Understanding the interaction between intention and the remaining motor preparatory processes is pivotal in optimising existing hybrid brain-machine interfaces [18], [19], especially those driving limb prostheses, or robots [20]–[23]. The central question of how the conscious motor intention is connected to complex motor programs still remains unclear. In the present study, we investigated the changes of consciously intended movement aims in the motor preparation phase and their motor outcome using kinetic data and MEP induced by transcranial magnetic stimulation (TMS). We demonstrate the presence of conscious intention in motor preparation prior to movement. Intention, in this sense, is the conscious origin of goal-directed movement preparation. Consequently, intentional behaviour is coupled to motor control processes.

**Figure 1:**
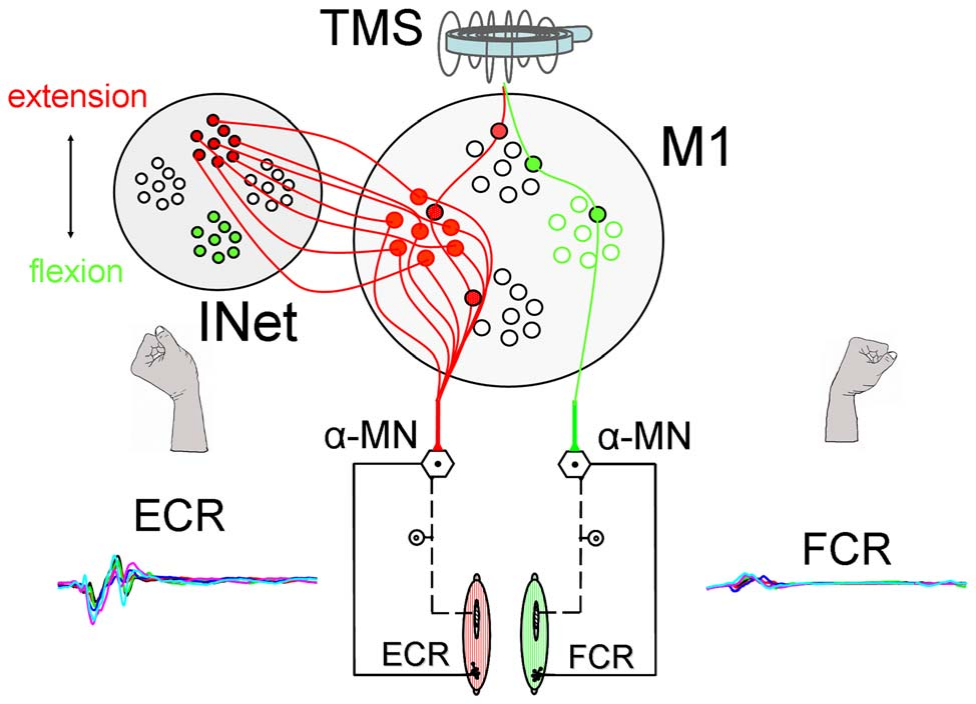
Model of Coupling Intention Network to Motor Cortex. Pyramidal cell potentials (M1), with different input intensity from the intention network (INet), were elevated transcranially by a magnetic impulse in parallel. In the case of the figure above, a TMS impulse and an input from the INet, simultaneously results in a depolarisation of some pyramidal cells (ECR red). We achieved stronger MEP responses when a simultaneous input from the TMS depolarised a large group of pyramidal cells, corresponding to a stronger input from the INet. The TMS impulse above the motor threshold simultaneously activated some of the antagonistic pyramidal cells (FCR green).

Using the method of TMS, it became possible more than two decades ago to stimulate the motor cortex transcranially and non-invasively, and to provoke non-specific movement in the limbs [24]. Even using modern TMS coils, however, it is impossible to specifically target either agonistic or antagonistic activation using TMS alone. Hence, TMS is generally omni-directional in nature, and simultaneously innervates both agonistic and antagonistic muscles. Motor intention is the conscious initialising stage in goal directed voluntary movement of the following motor preparatory processes, like motor planning, motor programming and motor execution. In the present study, we hypothesised that a consciously intended motor preparation of a wrist extension combined with a minimally supra-threshold TMS, results in a salient goal-directed wrist extension movement. In contrast, a low intensity TMS, triggers a wrist flexion in participants concentrating on forming a conscious motor intention to flex the wrist, with the same stimulation intensity and stimulation side at the primary motor brain for both muscle simultaneously. To test this hypothesis, we asked subjects to randomly select and concentrate on movement aims (wrist flexion/extension), and then elicited minimally supra-threshold TMS. We could show that intentional motor preparation was able to convert unspecific and weak TMS activation to direction-specific, strong MEPs and strong wrist movements. This demonstrates that conscious goal directed motor intention coins the residual motor preparatory processes in the motor cortex by an intention-conveying network (INet). These motor intentions, apparently, have effects on the excitability of the cortical motor neurons (Figure 1), comparable to those seen during actual motor performance.

The most outstanding position against this account represents Wegner (2003) [25], who generally challenged the idea of conscious will, before movement execution, and hypothesised that the perception of an intention is constructed in hindsight, after movement execution.

## Materials and Methods

### Ethics Statement

Ethical approval for the present study was given under the identifier A 201194 University Rostock – Germany in accordance with the Declaration of Helsinki. Participants were healthy uninjured sport students and staff members, who were informed of the investigation and gave written consent to participate in the investigation.

### Subjects

14 subjects took part, with an average age of 30.3±9.2 years. The subject's handedness was defined via a hand-score-test [26] – and only right handed subjects were chosen. We discarded two from the 14 participants because of difficulty in full relaxation in the baseline condition (BC). All participants had the opportunity to familiarise themselves with the experiment and with the treatment.

### Setting

Subject's heads were comfortable seated in an upright position, with their head positioned on a chin-forehead rest. Their left forearm was hanging in a relaxed position, between supination and pronation, with the elbow joint comfortably extended. The TMS-coil was held in a fixed position via a mechanically adjustable arm (Manfrotto Feltre, Italy) during the whole experiment. The head was fixed with a belt around the occiput at the forehead-rest to minimise relative coil-to-head movement. The coil position over the head was optimised to yield the lowest motor thresholds of both recorded muscles (see below). A no-intention condition served as a baseline condition (BC) for the MEPs. In this baseline condition, no motor preparation process took place and the TMS was triggered by the examiner. The stimulation site over the motor cortex was optimised for wrist muscle activation, using EMG and acceleration measurement to control evoked muscle potentials, and then kept constant throughout the entire experiment.

### Kinetic measurement and recording

Kinetic data of the wrist movement were sampled with a 1D–accelerometer (Biovision, Wehrheim, Germany), with a range of±5 g, simultaneously with surface electromyography (EMG) data. The device was fixed by a rigid frame on the dorsum of the left hand. All signals were sampled with a DAQ-Card 6024 with 12 bit amplitude resolution (National Instruments, Austin, Texas, USA) at a sampling rate of 10 kHz/channel. For data acquisition and further analysis, the signal processing program DIAdem (National Instruments, Austin, Texas, USA) was used.

### Protocol

Volunteers were asked to develop an intention to move the left hand (flexion or extension), as strong and fast as possible, and to trigger the TMS with the right index finger if the urge to move was greatest before any overt motor output at the wrist. Muscle activity was controlled by electromyography (EMG) online. A minimum of 10 seconds was required between each intention buildup. A typical, 25 stimulations were applied for each condition, extension (EXT), flexion (FLEX), and with no intention as the baseline condition (BC), in a random order. Trials with EMG pre-activity of more than 100 µV in a time-window of 100 ms before the TMS stimulus were discarded. Therefore, overall, between 37–65 valid data sets for each subject could be used with 15.1±4.8 valid trials in BC, 17.2±5.4 valid trials in FLEX and 19.3±3.9 valid trials in EXT. The total stimulation period lasted for a total of 10 to 15 minutes in each condition.

### Physiological methods

TMS elicited motor-evoked potentials were recorded with a differential amplifier (Biovision Wehrheim, Germany - input resistance of 10 GΩ, bandwidth of 1–1000 Hz). An amplification gain (500–1000×) was chosen to optimally adjust for individual electrode-skin-interface conditions. EMG raw signals from the extensor carpi radialis muscle (ECR), and the flexor carpi radialis muscle (FCR) were high-pass filtered with a digital 2nd order Butterworth filter [27] with a cut-off frequency of 5 Hz. Reflex responses were registered with Ag–AgCl cup electrodes (Hellige Baby-Electrodes; GE Medical Systems, Milwaukee, USA) with an electrode surface area of 3 mm^2^, placed at inter-electrode distances of 1 cm longitudinally over the belly of the wrist muscles. The reference electrode was fixed at the acromion process. The skin was cleaned with alcohol and any hair was removed before the electrode application. The skin impedance was generally lower than Z = 10 kΩ[28] at 30 Hz. To ensure good contact between the skin and an electrode, an electrode gel (Parker Laboratories, Fairfield, USA) was used. Electrodes and twisted cables were fixed with collodion C_6_H_8_(NO_2_)O_5_ and self-adhesive tape on the skin [29]. Muscle relaxation was continually monitored over the whole experiment using EMG. MEP responses were rejected offline whenever surface EMG activity became apparent (greater 100 µV - 100 ms time-window before TMS).

### Magnetic Stimulation

**Figure 2:**
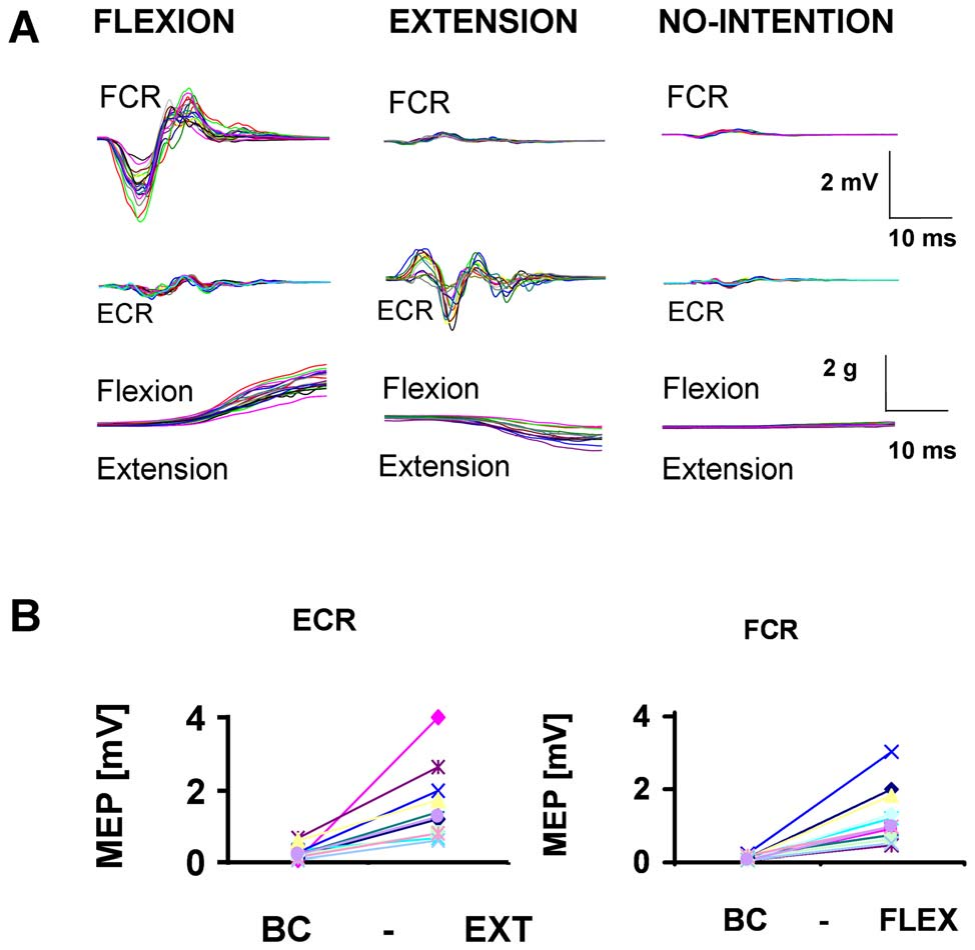
Rawdata and Effects of Intention on Motor Potentials and Kinetics. Representative trials of one subject (A) illustrates the MEP of the flexion directed movement (on the left side) with the flexor activities (top), the extensor activities (middle), and the time course of the wrist acceleration in the bottom graph. The columns represent the wrist flexion intention (left) and the wrist extension intention condition (middle) and the no-intention condition (BC right). All MEPs have the same scale-units. The acceleration of the left hand dorsum and electromyography (EMG) from the flexor carpi radialis (FCR) and extensor carpi radialis muscles (ECR) of the left hand were recorded for the kinetics of the wrist. (B) Mean values for the intention condition and the baseline condition with the ECR (intended extension) on the left and the FCR (intended flexion) on the right side are shown for each subject.

Transcranial magnetic stimulation was performed using pulsed magnetic fields. An R30 MagPro with MagOption (MagVenture, Skovlunde Denmark - formerly Medtronic) magnetic stimulator and an MMC-140 parabolic coil were used. The stimulator generated biphasic symmetric pulses (duration 290 µs). The coil was placed just behind the vertex over the right motor cortex, with the concave side placed on the surface of the skull. The parabolic coil was oriented so that the induced electric current flowed in an anterior-posterior direction over the right motor cortex. Stimulation intensities were adjusted (1–10 A/µs) to be just above the motor threshold to reach detectable MEP on the ECR and the FCR muscles of the left hand. Motor threshold was defined as the stimulus intensity that produced an MEP of 100 µV in 3 of 5 trials in the most insensitive muscle of the two. Intensities, just marginally above the motor threshold were chosen to minimise the perturbing effects of the stimulation when producing the strongest modulation. In our experiment, stimulation slightly lower than a 1.1× threshold turned out to be that best suited. Figure 2A demonstrates examples of the MEPs obtained from one subject. The magnetic gradient, therefore, lies in the range of 50–70 A/µ, depending on individual excitability. All subjects showed higher MEP responses in the intention conditions compared to the BC condition in Figure 2 B.

### Statistics

The amplitudes of the 1D-acceleration and the MEPs were compared between an intended flexion (FLEX), an intended extension (EXT), and baseline condition trials with no intention (BC). Peak-to-peak values (MEP_pp_) of filtered surface EMG data of MEPs were used for analysis. Amplitudes are expressed as absolute values of individual average MEP from all stimuli obtained at under different conditions. This procedure yields an adequate method for the assessment of excitability. Data was tested to confirm normal-distribution using the Shapiro-Wilk Test. Comparisons were made between the conditions of FLEX, EXT, and of the stimulation with BC. To test for statistically significant differences of means, a two-tailed paired Student's t-test was used. The level of statistical significance was set at p<0.05. Values in the text are given as a mean±standard deviation (SD). The peak to peak values of the MEP were calculated as an indicator of the cortico-spinal excitability.

## Results

**Figure 3:**
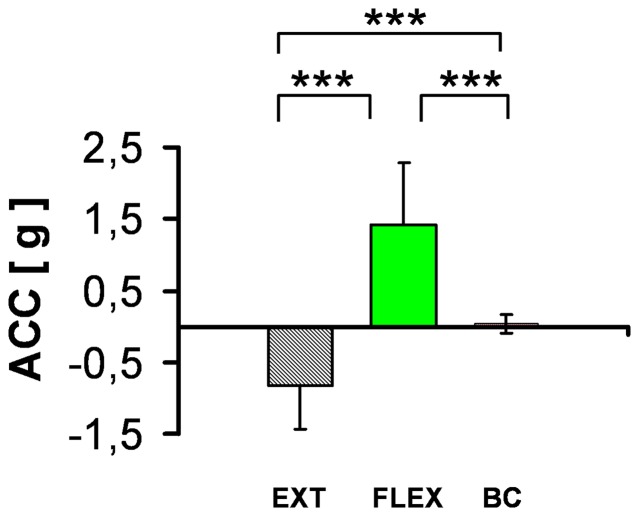
Effects of Intention on Kinetics. The acceleration of the wrist illustrates the kinetics produced by the extensor directed movement with the EXT activities (left) and the FLEX intension produced activities (middle). In the baseline condition (right bar of the diagram), only small omni-directed movements could be observed. Differences between the conditions were tested (with * p<0.05, ** p<0.01, *** p<0.001).

In all subjects, we found a strong intention-directed effect (Figure 3) on the movement. This was true both for the MEPs and the kinetic (acceleration) data: with an intended wrist extension, acceleration differed by - 0.820 g±0.615 g compared to BC with p<0.0003 (two-tailed paired t-test, df = 11 and t = 5.189); and with an intended wrist flexion by 1.426 g±0.868 g compared to BC with p<0.0002 (two-tailed paired t-test with df = 11 and t = 5.489).

In the current experiment, all subjects were able to generate clear goal-directed motor intention, which then could be triggered to an overt movement by a TMS impulse. In contrast, under full mental relaxation in the BC condition, omni-directed and weak activation of both agonists and antagonist was generated, as shown by the kinetic data (0.031 g±0.134 g).

**Figure 4:**
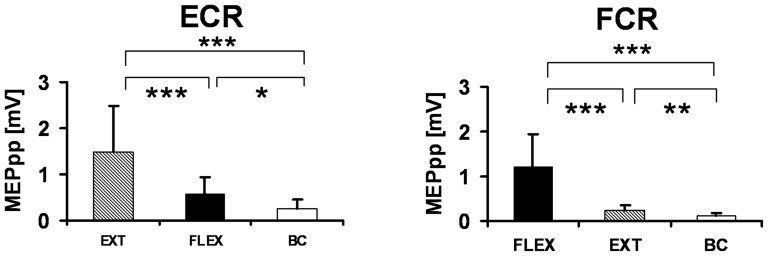
Effects of Intention on Motor Potentials. The averages for all subjects of the MEP-bar-graphs show typical effects of the movement intention on the MEP-amplitudes. The ECR (left side of the graph) had the greatest MEPs under the condition of an intended extension, which is the same for the FCR under the intended flexion of the wrist (right side of the graph). There were also small MEPs recorded, after TMS, in the baseline condition (* p<0.05, ** p<0.01, *** p<0.001).

The differences of the MEP amplitudes, between the antagonistic intention condition and the baseline condition, were also significant in all subjects (Figure 4, Table 1). Hence, the experiments show that there is a salient effect of motor intention and movement condition on the MEP of the flexor and extensor muscle, respectively.

**Table 1:** Motor Evoked Potentials of Intended Stimulation.

Condition	MEPpp [mV]			
	ECR M±SD		FCR M±SD	
FLEX	0.584	0.354	1.201	0.748
EXT	1.482	1.003	0.238	0.124
BC	0.245	0.199	0.107	0.061

The different experimental conditions of Flexion, Extension, and No-Intention (BC) resulted in different MEP-Amplitudes of the antagonistic muscle pair of the forearm (FCR and ECR). The Amplitudes of the EMG-data were not normalised. Mean (M) and standard deviations (SD) are presented.

Again, recording EMG at flexor and extensor muscles under conditions of motor intention INT (flexion/extension) vs. no motor intention (BC), TMS alone, with intensity minimally above the motor threshold, elicited small, unpredictable and omni-directed responses in the different muscle groups. In all subjects, the differences of the MEP amplitudes between the intention conditions of wrist extension, wrist flexion and the baseline condition with no intention, were found to be significant (analysis of variance (ANOVA) for repeated measures for the ECR results as follows: p<0.0001, F (2, 33)  = 12.58 and for the FCR with p<0.0001 and F (2, 33)  = 22.20 for all conditions). All data is presented with a mean and SD in Table 1, and t-test results are compared between all conditions in Table 2.

**Table 2:** T-Test of Motor Evoked Potentials under Different Conditions.

	ECR		FCR	
Condition	EXT	- FLEX	EXT	- FLEX
df = 11	t = 4.341	p = 0.001	t = 4.588	p = 0.001
Condition	FLEX	- BC	FLEX	- BC
df = 11	t = 3.020	p = 0.011	t = 5.318	p = 0.001
Condition	EXT	- BC	EXT	- BC
df = 11	t = 4.350	p = 0.001	t = 3.433	p = 0.006

The different experimental conditions of Flexion, Extension, and No-Intention (BC) resulted in different MEP-Amplitudes of the antagonistic muscle of the forearm (FCR and ECR). The T-Test values of the MEP-data, degrees of freedom, and p-values are presented (* p<0.05, ** p<0.01, *** p<0.001).

## Discussion

The experimental investigation of the relationship of intention and movement execution could only partly benefit with a traditional philosophical discourse on intention in voluntary movement [16]. Since the pioneering experiments of Kornhuber and Deeke [30], it is known that brain activity occurs before any movement execution and that these brain potentials can be measured in humans. These experimental results have stimulated an even ongoing controversial debate about the role of these brain potentials and their consequences for voluntary movement [1]–[3], [31]–[33]. Furthermore, Wegner [25] generally challenged the experience, by questioning, whether the conscious perception of an intended movement is generated before movement execution, or whether this attribution is constructed in hindsight. Understanding the intentional processes in voluntary movement generation is an important issue in many scientific research areas.

An emerging focus of research has developed in recent decades, that deals with intentional processes of reach-to-grasp and grasping movements. Most particularly, an area has emerged, which considers the influence of intention and movement goals on the kinematic outcome.

Furthermore, studies in primates have shown which cortical areas were active in reach-to-grasp movement in the preparatory motor phase [34]. These experiments typically examine the neuronal activity in instructed-delay-tasks, with the assumption that the preparatory activity shares common brain areas with the early post-GO activity in normal reaction-time-tasks. Differences between the two conditions were prominent in the PMd neurons. The functional role of PMd neurons still remains unclear due to the inhibition of movement in the delayed movement task [35], [36].

Many facets between a single neuron activity and the social influence on the intention of people acting have been investigated. The influence of social interaction has recently been examined for the generation of actions and movements [37]. These social intentions deal with the *perception of the intentions* of a third person. This is in contrast to our experiment, where we consider the *intention to move* by the person himself/herself. Also, environmental conditions, for example, affect visual information in specific characteristics such as the size of objects, but also the information in abstract form, such as the labeling of objects, has an influence in the process of motor preparation [38]. While the above mentioned studies have examined dominant external effects on motor execution, in the present study, internal effects have an influence on movement generation, and in this particular case a specific intention.

Conscious intentional processes in humans were detected with a direct electrical stimulation of the brain by Fried et al. [6], in the supplementary motor area (SMA), and by Desmurget et al. [39] in the parietal cortex, and with an fMRI by Boussaoud [4] in the SMA, and in the more caudal part of the dorsal premotor area (PMDc), and in attention to intention from Lau et al. [8] in the intraparietal sulcus (IPS). In contrast, many fMRI studies show an overlap of brain activity, during motor imagery of the above mentioned cortical areas (for review see [40], [41]). Consequently, it is not clear how to dissociate these two brain states, with the methods of brain imaging, if at all possible. A recently published work [42] comes to the conclusion that there is no difference between imagery and intention. Therefore, the hypothesis stands that same brain circuits are involved in imagery and motor preparation.

However, psychological models of motor preparation come perhaps the nearest to that process of intention we investigated in our experiment. Castiello and Jeannerod [43] and Castiello et al. [44] dealt with the awareness of movement correction and examined the time difference between motor awareness and motor response. Later Jeannerod [45] provided a *model of self generated action* and, in this model of motor representation, he divided the process of motor preparation into different parts, whereby in a sequential view, the intention stands at the beginning of the motor preparation, followed by motor planning, motor programming and motor execution. From this view, motor intention is a stage in motor preparation before movement execution.

The results of the present study show that *conscious, voluntary intention* coins the kinematic direction of targeted wrist movement elicited by TMS. With an intention (INT) of a wrist flexion movement, the kinetic data shows a wrist flexion, and correspondingly, evoked MEP responses that were significantly larger in the flexor than the extensor muscles; the opposite was true for an extension intention where the MEP were significantly larger in extensor than in the flexor muscles. Hence, a mere conscious intention, of a purposeful movement, is able to modulate movement direction of an otherwise unspecific TMS-triggered movement output. In an earlier experiment, we could show that the net excitability of the motor network is modulated during motor execution, by environmental demands [46], and the intentional activation of its motor neurons, at the time of the TMS impulse, which include both firing neurons and those remaining inferior.

Several authors have already shown that the TMS-induced MEPs were unspecifically elevated during active muscle contraction [47]–[52], and also during mental imagery of movement [10], [13], [36]–[46], [53]–[62]. Further, Bonnard et al. [63] were able to show that an intention to resist vs. an intention to assist a perturbation tune of TMS-evoked N100 EEG amplitudes of the motor cortex area. These studies showed that the strength of the voluntary drive modulates cortico-spinal excitability *during* the movement phase, or further increased the kinetic response with a single-pulse TMS application, again *during* directed movement execution [64]. It was also shown that TMS in the movement preparation phase can influence the selection probability of motor programs [65], [66] and also affects the reaction time properties [67], [68].

According to previous studies [13], [69], we could show that motor cognition tunes the cortico-spinal exitability. But more specifically, with the motor intention paradigm, all subjects were able to produce low intensity TMS directed motor responses in either flexor or extensor muscles of the wrist depending on the chosen intention. In the study of Gandevia & Rothwell [69], where the participants had the task to focus on a specific muscle, they were unable to influence the muscle of the forearm, but the intrinsic hand muscles sometimes showed small responses by transcranial electrical stimulation. In the case of the motor imagery experiment by Fadiga et al. [13], they found an imagery effect of a forearm flexion on the biceps brachii muscle, and no response of an extension-imagery on the same muscle. Likewise, these effects were small and no directed motor responses were reported from this experiment.

A very recent publication on cognitive processes before voluntary movement [70] shows the problems in understanding the emergence of conscious intention. These are nicely illustrated by Desmurget's [71] comments on that paper, in which he provides three competing hypotheses for this problem; two of which have a serial character, and one hypothesis further shows a parallel property of movement preparation and intention genesis. Desmurget [71] assigns to Schneider et al. [70] a serial hypothesis of a "planning *then* intention" interpretation of motor intention and a second controversial serial hypothesis of "intention *then* planning" as an everyday life hypothesis. Much more interesting is the third hypothesis, arguing with a *parallel* development of movement planning *and* a simultaneous emergence of conscious intention. Basically, Cisek and Kalaska [72] find some evidence for a model that brain processes do run in parallel, including decision processes, and action selection. When discussing serial vs. parallel computing in motor preparation, it is important to bear in mind that parallel processes cannot really have a strong chronological order which is due to the inherent matching problems between parallel processes. This, in turn, prompts to question experiments based on *subjective* time perception, since parallel vs. sequential processes cannot be distinguished in these objectively [31]. Furthermore, one must assume that numerous processes in the brain are not working strictly serially, but have a significant recursive kernel [73] in a sparse distributed network.

In the debate about conscious intention, Desmurget and Sirigu [74] made a plausible distinction between an “urge to move” by direct electrical stimulation of the mesial-precentral area [75] and the “wanting to move”. Desmurget and Sirigu [39] used a direct electrical stimulation technique for stimulating the inferior parietal lobe, from patients undergoing brain surgery, which resulted in a less specific “wanting to move”, yet presumably, without actual motor plan. This, in turn, demonstrates that intention must be understood as a process with some temporal extension, and cannot be an infinitesimally brief process or pulse. Comprehension of motor intention as a process, and not a serial impulse, might help us to better understand how consciousness works in voluntary movement preparation.

We propose here that putative intention-conveying networks (INet) serve to increase the excitatory drive on motor neurones in the primary motor cortex (see Figure 1). Under such conditions of raised excitatory input from the INet, bringing neurons close to threshold, a TMS impulse is able to induce preferential firing of those cells, with a specific cortical projection to the spinal motor neurons, which obey the intended movement direction. The goal-directed excitation, in turn, is likely to favour the recruitment of specific spinal motor neuron pools. This modulation, effected by the INet, may be especially important, in shaping the evoked direction dependent MEPs. Since TMS only acts on superficial cortical areas, our data does not rule out an involvement of the sub-cortical structures on the regulation of agonistic and antagonistic muscle activation, or suppression during movement intention; instead, the data supports the notion of an intention network, crucially modulating primary motor cortex activity.

However, the novelty of our study is, that volition, and indeed an intention element, *prior* to movement, resulting in direction specificity, and this, in turn, could not be demonstrated up to now. Moreover, we can state by evidence that consciousness of a movement intention comes prior to movement execution. This clearly shows that the experience of willing is obviously perceived before the movement execution. This result of our study stands in contrast to Wegner's [25] interpretation of the consciousness of will in the case of voluntary movement.

## Conclusion

Motor intention (intention in action) [16] describes a process of motor preparation without executing an overt movement. In our study, we explored the link between motor intention in the movement preparatory phase and the motor outcome. The experiments present evidence that the excitability of the agonistic motor system is significantly enhanced when subjects develop an intention to move. The opposite was true for the antagonistic movement direction and muscles. The results presented indicate that the excitatory cortico-spinal drive is enhanced during directed motor intention. The data shows that movement intention induced during the enhancement of the cortico-spinal pathway was significantly greater than in the no-intention condition, which argues for the movement-specific modulation of cortico-spinal excitability. The results support the hypothesis that conscious intention to move induces the enhancement of target-specific motor circuits prior to overt movement execution.
